# Zn(ii)2,9-dimethyl-1,10-phenanthroline stimulates cultured bovine aortic endothelial cell proliferation†

**DOI:** 10.1039/d0ra06731h

**Published:** 2020-11-20

**Authors:** Takehiro Nakamura, Eiko Yoshida, Takato Hara, Tomoya Fujie, Chika Yamamoto, Yasuyuki Fujiwara, Fumihiko Ogata, Naohito Kawasaki, Ryo Takita, Masanobu Uchiyama, Toshiyuki Kaji

**Affiliations:** Faculty of Pharmaceutical Sciences, Tokyo University of Science 2641 Yamazaki Noda 278-8510 Japan; Faculty of Pharmacy, Kindai University 3-4-1 Kowakae Higashi-Osaka 577-8502 Japan; Faculty of Pharmaceutical Sciences, Toho University 2-2-1 Miyama Funabashi 274-8510 Japan; School of Pharmacy, Tokyo University of Pharmacy and Life Sciences 1432-1 Horinouchi Hachioji 192-0392 Japan; Graduate School of Pharmaceutical Sciences, The University of Tokyo 7-3-1 Hongo Bunkyo-ku 113-0033 Japan; Advanced Elements Chemistry Research Team, RIKEN Center for Sustainable Resource Science, Elements Chemistry Laboratory, RIKEN 2-1 Hirosawa Wako 351-0198 Japan

## Abstract

Vascular endothelial cells cover the luminal surface of blood vessels in a monolayer. Proliferation of these cells is crucial for the repair of damaged endothelial monolayers. In the present study, we identified a zinc complex, Zn(ii)2,9-dimethyl-1,10-phenanthroline (Zn-12), that stimulates the proliferation of bovine aortic endothelial cells in a culture system. No such stimulatory activity was observed for the ligand alone or in combination with other metals; however, the ligand combined with iron weakly stimulated the proliferation, as evidenced by the [^3^H]thymidine incorporation assay. Inorganic zinc weakly but significantly stimulated proliferation, and intracellular accumulation of zinc was similar between inorganic zinc and Zn-12 treatment, suggesting that the mechanisms by which Zn-12 stimulates vascular endothelial cell proliferation contain processes that differ from those by which inorganic zinc stimulates proliferation. Although expression of endogenous fibroblast growth factor-2 (FGF-2) and its receptor FGFR-1 was unchanged by Zn-12, both siRNA-mediated knockdown of FGF-2 and FGFR inhibition partly but significantly suppressed the stimulation of vascular endothelial cell proliferation by Zn-12, indicating that the zinc complex activates the FGF-2 pathway to stimulate proliferation. Phosphorylation of ERK1/2 and MAPKs was induced by Zn-12, and PD98059, a MEK1 inhibitor, significantly suppressed the stimulatory effect of Zn-12 on vascular endothelial cell proliferation. Therefore, it is suggested that Zn-12 activates the FGF-2 pathway *via* activation of ERK1/2 signaling to stimulate vascular endothelial cell proliferation, although FGF-2-independent mechanisms are also involved in the stimulation. Zn-12 and related compounds may be promising molecular probes to analyze biological systems of vascular endothelial cells.

## Abstract 2

Stimulation of vascular endothelial cell proliferation by Zn-12 can be mediated by the ERK1/2 activation independently of the FGF-2-FGFR pathway. Additionally, there may be other pathways involved in the Zn-12 stimulation.

## Graphical abstract

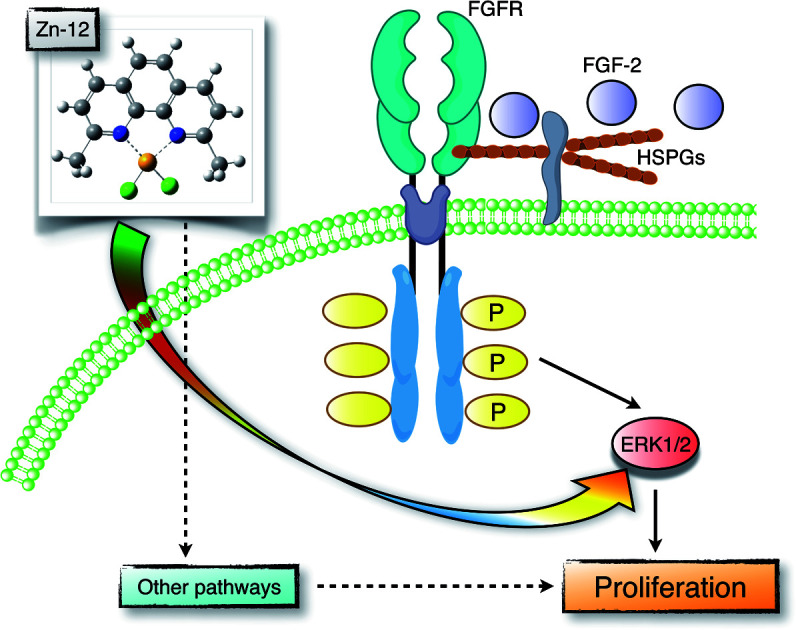

## Introduction

Vascular endothelial cells cover the luminal surface of blood vessels in a monolayer. The monolayers not only function as a barrier between the blood and the subendothelial matrix but also regulate the blood coagulation–fibrinolytic system by synthesizing and secreting prostacyclin,^1^ anticoagulant proteoglycans,^2–5^ and fibrinolytic plasminogen activators.^6^ It is postulated that the anticoagulant and fibrinolytic properties of vascular endothelial cells prevent the initiation and progression of vascular lesions, such as atherosclerosis.

Proliferation of vascular endothelial cells is crucial for the maintenance of the monolayers. Fibroblast growth factor-2 (FGF-2) is a growth factor that promotes endothelial cell proliferation in an autocrine fashion.^7,8^ FGF-2 has no signal sequence that directs its secretion *via* the normal secretory pathway.^9^ Therefore, it is believed that the growth factor mainly leaks from damaged endothelial cells and stimulates the proliferation of the cells near the site of damage. We found that inorganic zinc stimulates cultured vascular endothelial cell proliferation, depending on endogenous FGF-2, and promotes the repair of damaged cell layers.^10,11^

Bio-organometallics is a research strategy that uses organic–inorganic hybrid molecules, that is, organometallic compounds and metal complexes, as molecular probes to analyze biological systems.^12^ We have previously analyzed intracellular signaling pathways that mediate vascular endothelial cell functions, revealing that a copper complex and organoantimony compounds induce the transactivation of metallothionein, a cytoprotective protein, by activating the MTF-1–MRE and Nrf2–ARE pathways.^13–15^ Additionally, using organic–inorganic hybrid molecules as molecular probes, it was found that the expression of syndecan-4, a transmembrane-type of heparan sulfate proteoglycans, can be induced by the activation of the hypoxia-inducible factor-1α/β and p38 MAPK pathways.^16,17^

Based on our previous studies stated above, we hypothesized that there are zinc complexes that can stimulate vascular endothelial cell proliferation. We propose that such complexes, used as molecular probes, will contribute to unravelling the mechanisms underlying endothelial cell proliferation. The purpose of the present study was thus to identify a zinc complex from a library of zinc complexes that strongly stimulates vascular endothelial cell proliferation and to investigate some of the mechanisms underlying the stimulation.

## Experimental

### Materials

Bovine aortic endothelial cells were purchased from Cell Applications (San Diego, CA, USA). Dulbecco's modified Eagle's medium (DMEM) and Ca^2+^- and Mg^2+^-free phosphate-buffered saline (CMF-PBS) were obtained from Nissui Pharmaceutical (Tokyo, Japan). Fetal bovine serum (FBS) was purchased from Biosera (Kansas, MO, USA). Cytotox 96® Non-Radioactive Cytotoxicity Assay, a lactate dehydrogenase kit, was purchased from Promega (Madison, WI, USA). The HiPerFect Transfection Reagent and QIAzol lysis reagent were obtained from Qiagen (Hilden, Germany). The bicinchoninic acid protein assay kit was purchased from Thermo Fisher Scientific (Waltham, MA, USA). [Methyl-^3^H]thymidine (MT-6039) was purchased from Moravek Biochemicals (Brea, CA, USA). PD98059 (a MEK1 inhibitor) was purchased from Enzo Life Sciences (Farmingdale, NY, USA). PD161570, an FGF receptor-1 (FGFR1) inhibitor, was purchased from Cayman Chemical (Ann Arbor, MI, USA). Horseradish peroxidase-conjugated anti-rabbit (#7074) and anti-mouse (#7076) IgG antibodies, rabbit monoclonal antibodies against basic FGF (19A9), rabbit polyclonal antibody against FGFR1 (#3472), p44/42 MAPK (ERK1/2) (#9102), phospho-p44/42 MAPK (ERK1/2) (#9101), p38 MAPK (#9102), and phospho-p38 MAPK (#9211) were obtained from Cell Signaling Technology (Beverly, MA, USA). Mouse monoclonal antibodies against JNK (D-2) (sc-7345) and phospho-JNK (G-7) (sc-6254) were obtained from Santa Cruz Biotechnology (Santa Cruz, CA, USA). Mouse monoclonal antibodies against β-actin and recombinant human basic FGF were obtained from Fujifilm Wako Pure Chemical Industries (Osaka, Japan). A high-capacity cDNA reverse transcription kit was purchased from Applied Biosystems (Foster City, CA, USA). Gene Ace SYBR qPCR Mixα was obtained from Nippon Gene (Tokyo, Japan). Other reagents were obtained from Nacalai Tesque (Kyoto, Japan).

### Synthesis of organic–inorganic hybrid molecules

The zinc complexes used in this study were synthesized as described previously^18^ with some modifications and identified by elemental analysis. Complexes of 2,9-dimethyl-1,10-phenanthroline (DMP) with manganese (Mn-DMP), iron (Fe-DMP), cobalt (Co-DMP), nickel (Ni-DMP), copper (Cu-DMP), cadmium (Cd-DMP), mercury (Hg-DMP), and lead (Pb-DMP) were synthesized as described previously.^18^ In this procedure, metal ions and ligands were dissolved in a stoichiometric ratio of 1 : 1 in a suitable solvent to crystallize metal complexes. As free zinc ions were removed using filtration, we hypothesized that the experiments described below were performed using none or trace amounts of inorganic zinc as an impurity. Results of the elemental analysis indicated that there were little or no organic impurities. The data are shown in Table S1.† Thus, the results obtained in the present study can be regarded as the effects of synthesized metal complexes.

### Cell culture

Vascular endothelial cells were cultured in DMEM supplemented with 10% FBS in 100 mm dishes at 37 °C in a humid atmosphere of 5% CO_2_ until confluent. The cells were then transferred to 24-well or 6-well culture plates at 1 × 10^4^ cells per cm^2^ and cultured in fresh DMEM supplemented with 10% FBS for 24 h (growing cultures). The growing cultures were used for subsequent experiments.

### Cell proliferation assay

The proliferation of growing cultures of vascular endothelial cells was evaluated by the incorporation of [^3^H]thymidine into the acid-insoluble fraction of the cells.^10^ Briefly, growing cultures in 6-well plates were treated with different zinc complexes (Zn-1, Zn-2, Zn-3, Zn-4, Zn-5, Zn-6, Zn-7, Zn-8, Zn-9, Zn-10, Zn-11, Zn-12, Zn-13, Zn-14, Zn-15, Zn-16, Zn-17, Zn-18, Zn-19, Zn-20, Zn-21, Zn-22, Zn-23, Zn-24, Zn-25, Zn-26, Zn-27, Zn-28, Zn-29, Zn-30, Zn-31, and Zn-32), inorganic zinc (ZnSO_4_ or ZnCl_2_), or the DMP ligand with or without a metal (DMP, Mn-DMP, Fe-DMP, Co-DMP, Ni-DMP, Cu-DMP, Cd-DMP, Hg-DMP, or Pb-DMP) at 10, 20, 30, or 50 μM each for 24 h in serum-free DMEM for 24 h after treatment with or without an inhibitor of FGFR1 (PD161570) at 1 μM or that of MEK (PD98059) at 0.1 or 0.5 μM for 4 h. The cells were labeled with [^3^H]thymidine at 100 kBq mL^−1^ during the last 4 h of the treatment. Cell homogenates were prepared by sonication (Ultrasonic Homogenizer UX-050; Mitsui Electric, Noda, Chiba, Japan), and the incorporation of [^3^H]thymidine into the 5% trichloroacetic acid-insoluble fraction of the cell homogenates was determined by liquid scintillation counting (TRI-CARB2800TR, PerkinElmer, Waltham, MA, USA). A portion of the cell homogenates was used for the determination of the DNA content by fluorometric assay.^19^

### Nonspecific cell damage

Growing cultures of vascular endothelial cells in 24-well plates were treated with ZnSO_4_ (10 μM) or Zn-12 (10, 20, 30, or 50 μM) for 24 h in serum-free DMEM. Nonspecific cell damage was evaluated by the leakage of lactate dehydrogenase from the cells into the conditioned medium, using the Cytotox 96® kit. Because this enzyme leaks from damaged cells, its leakage is a reliable indicator of cell damage. Enzyme activity was measured by conversion of a tetrazolium salt into a red formazan product; the amount of the red color produced after conversion was proportional to the amount of leaked lactate dehydrogenase from damaged cells or, in other words, to the number of damaged cells. As the results obtained using this method are consistent with morphological evaluation of cells, this kit has been widely used for cytotoxicity assays.^18,20–23^ Furthermore, we assessed the cytotoxicity of Zn-12 by morphological observations and by evaluating lactate dehydrogenase leakage, in this study.

### Small-interfering RNA (siRNA) transfection

Growing cultures of vascular endothelial cells were transfected with FGF-2 siRNA at 37 °C for 4 h in serum-free DMEM. The sequences of the sense and antisense strands of the siRNAs targeting bovine FGF-2 were as follows: 5′-GAAAGAAGAUGGAAGAUUACUTT-3′ (sense) and 5′-UAAUCUUCCAUCUUCUUUCAUTT-3′ (antisense). A nonspecific sequence was used as a control (Qiagen, Valencia, CA, USA). Transfection was performed using the HiPerFect Transfection Reagent according to the manufacturer's protocol. Briefly, annealed siRNA duplex (44 pmol mL^−1^) and HiPerFect Reagent (7.4 μL mL^−1^) were dissolved in Opti-MEM and incubated for 5 min at room temperature. After transfection, the medium was replaced with fresh DMEM supplemented with 10% FBS and incubated at 37 °C for 20 h. The medium was then discarded, and the cells were washed twice with serum-free DMEM. The cells were treated with or without Zn-12 (30 μM) for 24 h, and the proliferation was evaluated by the [^3^H]thymidine incorporation assay as described above.

### Intracellular accumulation of zinc and iron

Growing cultures of vascular endothelial cells were prepared in 6-well plates and treated with ZnSO_4_, ZnCl_2_, Zn-12, or Fe-DMP at 30 μM each for 24 h in fresh serum-free DMEM. The zinc or iron content was determined by inductively coupled plasma mass spectrometry (NexION 300S, PerkinElmer), as previously described.^18^

### Expression of FGF-2 and FGFR1

Growing cultures of vascular endothelial cells in 60 mm dishes were treated with Zn-12 at 5, 10, 20, and 30 μM for 24 h. The medium was then discarded, and the cells were washed twice with 1 mL of CMF-PBS. The cell layer was lysed in sodium dodecyl sulfate (SDS) sample buffer (50 mM Tris–HCl buffer solution containing 2% SDS and 10% glycerol at pH 6.8) and incubated at 95 °C for 3 min. Protein concentrations were determined using a bicinchoninic acid protein assay reagent kit. 2-Mercaptoethanol and bromophenol blue (1.67% each) were added to the samples (10 μg protein) and incubated at 95 °C for 3 min. Cellular proteins were separated by SDS-polyacrylamide gel electrophoresis using a 15% separating gel and a 4% stacking gel and transferred onto a polyvinyl difluoride membrane (Immobilon-P; Merck KGaA, Darmstadt, Germany) at 2 mA cm^−2^ for 1 h. The membranes were blocked with 5% skim milk in 20 mM Tris–HCl buffer solution (pH 7.5) containing 150 mM NaCl and 0.1% Tween 20 for 1 h and then incubated overnight with anti-FGFR1 (#3472) antibody (1 : 1000), anti-FGF-2 (19A9) antibody (1 : 1000), anti-phospho-ERK1/2 antibody (1 : 1000), anti-phospho-p38 MAPK antibody (1 : 1000), anti-phospho-JNK antibody (1 : 1000), anti-ERK1/2 antibody (1 : 1000), anti-p38 MAPK antibody (1 : 1000), anti-JNK antibody (1 : 1000), or anti-β-actin antibody at 4 °C. The membranes were washed with 20 mM Tris–HCl buffer solution (pH 7.5) containing 150 mM NaCl and 0.1% Tween 20 and incubated with horseradish peroxidase-conjugated anti-rabbit IgG antibody (1 : 5000) or anti-mouse IgG antibody (1 : 5000) for 1 h at room temperature. Immunoreactive bands were visualized by enhanced chemiluminescence using western blot detection reagents (Chemi-Lumi One; Nacalai Tesque, Kyoto, Japan) and detected with a LAS3000 Imager (Fujifilm, Tokyo, Japan).

For detection of the FGF-2 and FGFR1 mRNAs, growing cultures of vascular endothelial cells were treated with Zn-12 at 5, 10, 20, and 30 μM for 8 h in serum-free DMEM. The cells were washed twice with CMF-PBS and lysed using the QIAzol lysis reagent. One-quarter volume of chloroform was added to the cell lysate and centrifuged at 12 000 × *g* for 15 min at 4 °C. The supernatant was transferred into a fresh tube and mixed with the same volume of 70% ethanol. After incubation for 10 min on ice, the mixture was centrifuged at 12 000 × *g* for 10 min at 4 °C, and the supernatant was carefully removed. The pellet was mixed with 1 mL of 70% ethanol and centrifuged at 12 000 × *g* for 5 min at 4 °C. The supernatant was carefully removed. The pellet containing total RNA was dried at room temperature for 10 min and mixed with 20 μL of RNase-free water. Complementary DNA was synthesized from the total mRNA using a High-Capacity cDNA Reverse Transcription kit. Real-time reverse transcription-polymerase chain reaction was performed on a StepOnePlus system (Thermo Fisher Scientific) using the Gene Ace SYBR qPCR Mixα, 0.1 μM of each primer, and 1 ng μL^−1^ cDNA. Expression levels of FGF-2, FGFR1, and β_2_-microglobulin (B2M) were quantified by the comparative *C*_t_ method. Fold changes in the expression of FGF-2 and FGFR1 mRNAs were assessed by normalizing their intensity value to that of B2M. The following primer pairs were used: bovine FGF-2, 5′-AACCGTTACCTTGCTATG-3′ (forward) and 5′-CCCAGTTCGTTTCAGTGCC-3′ (reverse); bovine FGFR1, 5′-ACATTGAGGTGAACGGGAGTAAG-3′ (forward) and 5′-GAGTGATGGGAGAGTCCGATAGAG-3′ (reverse); bovine B2M, 5′-CCATCCAGCGTCCTCCAAAGA-3′ (forward) and 5′-TTCAATCTGGGGTGGATGGAA-3′ (reverse).

### Statistical analysis

Data were analyzed for statistical significance by analysis of variance (one-way ANOVA) and Student's *t*-test or Tukey's method as appropriate. *p* values less than 0.05 were considered to indicate statistically significant differences.

## Results

**Fig. 1:**
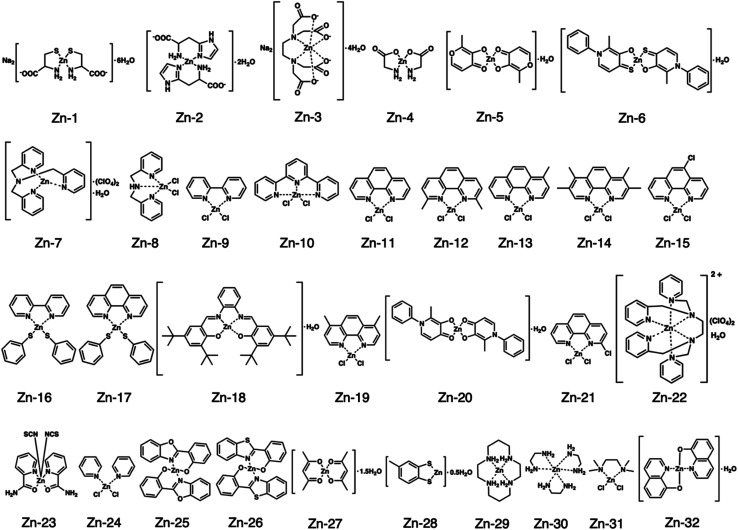
Structures of all zinc complexes used in this study.

**Fig. 2:**
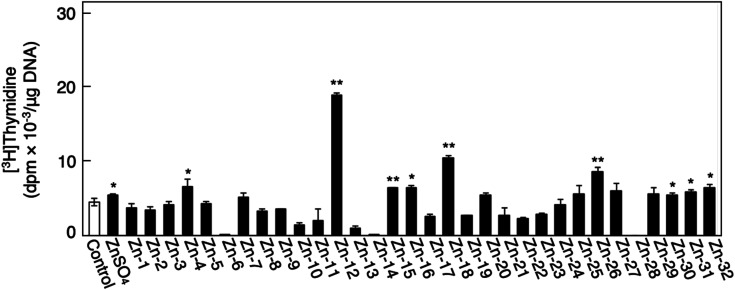
The incorporation of [^3^H]thymidine into the acid-insoluble fraction of vascular endothelial cells after treatment with inorganic zinc or zinc complexes. Growing cultures of bovine aortic endothelial cells were treated with ZnSO_4_ or zinc complexes (20 μM each) for 24 h and labeled with [^3^H]thymidine during the last 4 h of the treatment. Values are means ± SE of four samples. *p < 0.05 *vs.* control; **p < 0.01 *vs.* control.

**Fig. 3:**
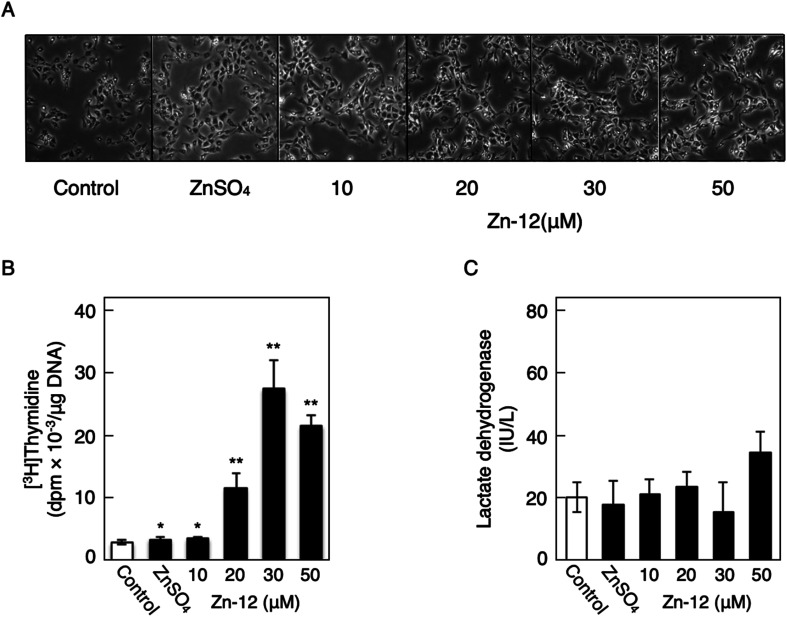
The stimulatory activity of Zn-12 on vascular endothelial cells without exhibiting cytotoxicity. Growing cultures of bovine endothelial cells were treated with ZnSO_4_ (10 μM) or Zn-12 (10, 20, 30, or 50 μM) for 24 h and labeled with or without [^3^H]thymidine during the last 4 h of the treatment. (A) Morphological observation. (B) The incorporation of [^3^H]thymidine into the acid-insoluble fraction of vascular endothelial cells after treatment with ZnSO_4_ or Zn-12. Values are means ± SE of four samples. *p < 0.05 *vs.* control; **p < 0.01 *vs.* control. (C) The leakage of lactate dehydrogenase from vascular endothelial cells into the medium after treatment with ZnSO_4_ or Zn-12. Values are means ± SE of four samples.

Fig. 1 shows the structures of all 32 zinc complexes used in this study. The compounds were assessed as stimulators of vascular endothelial cell proliferation. As shown in Fig. 2, inorganic zinc (ZnSO_4_) and 9 of the 32 zinc complexes (Zn-4, Zn-12, Zn-15, Zn-16, Zn-18, Zn-26, Zn-30, Zn-31, and Zn-32) significantly increased [^3^H]thymidine incorporation in growing cultures of vascular endothelial cells. The highest increase was observed for Zn-12, suggesting that this zinc complex is the strongest stimulator of vascular endothelial cell proliferation among the tested compounds. Morphologically, Zn-12 significantly increased the number of vascular endothelial cells without change in cell size (Fig. 3A) and the amount of incorporated [^3^H]thymidine in the cells in a concentration-dependent manner without increasing the leakage of lactate dehydrogenase (Fig. 3B and C). These results indicate that Zn-12 strongly stimulates vascular endothelial cell proliferation without nonspecific cell damage.

**Fig. 4:**
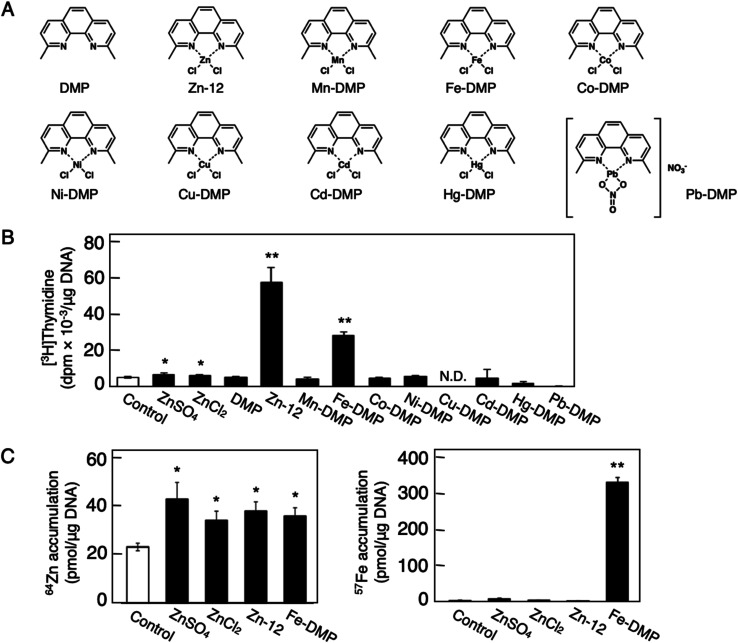
Role of zinc and DMP in the stimulatory effect of Zn-12 on vascular endothelial cell proliferation. Growing cultures of bovine endothelial cells were treated with ZnSO_4_ (10 μM), ZnCl_2_ (10 μM), DMP, Zn-12, Mn-DMP, Fe-DMP, Co-DMP, Ni-DMP, Cu-DMP, Cd-DMP, Hg-DMP, or Pb-DMP (30 μM each) for 24 h and labeled with or without [^3^H]thymidine during the last 4 h of the treatment. (A) Structure of DMP, Zn-12, Mn-DMP, Fe-DMP, Co-DMP, Ni-DMP, Cu-DMP, Cd-DMP, Hg-DMP, and Pb-DMP. (B) The incorporation of [^3^H]thymidine into the acid-insoluble fraction of vascular endothelial cells after treatment with ZnSO_4_, ZnCl_2_, or metal complexes. Values are means ± SE of four samples. *p < 0.05 *vs.* control; **p < 0.01 *vs.* control. N.D.: not detected. (C) The intracellular accumulation of zinc (left panel) and iron (right panel) in vascular endothelial cells after treatment with ZnSO_4_, ZnCl_2_, Zn-12, or Fe-DMP. Values are means ± SE of four samples. *p < 0.05 *vs.* control; **p < 0.01 *vs.* control.

The role of the coordinated zinc ion and the ligand in the stimulatory effect of Zn-12 on vascular endothelial cell proliferation was investigated by comparing the stimulatory effects of the ligand structure DMP, Zn-12, Mn-DMP, Fe-DMP, Co-DMP, Ni-DMP, Cu-DMP, Cd-DMP, Hg-DMP, and Pb-DMP (Fig. 4A). As described above, Zn-12 significantly increased [^3^H]thymidine incorporation. Fe-DMP also significantly increased [^3^H]thymidine incorporation, but the stimulatory activity was less than that of Zn-12. The other complexes tested, as well as DMP alone, did not show such activities (Fig. 4B). The intracellular accumulation of zinc was significantly increased after treatment with ZnSO_4_, ZnCl_2_, Zn-12, and Fe-DMP; however, the degree of the increase was almost the same among these compounds (Fig. 4C, left panel). These results suggest that the whole structure of Zn-12 (zinc ions and ligand) is required for the stimulation of vascular endothelial cell proliferation, with zinc being a specific metal that induces stimulation by interacting with the DMP structure as an intramolecular metal. Treatment with Fe-DMP significantly increased the accumulation of iron in the cells, but neither inorganic zinc nor Zn-12 caused such an increase (Fig. 4C, right panel), suggesting that the stimulatory effect of Zn-12 was not due to an increase in intracellular iron and that Fe-DMP also requires its whole structure to stimulate vascular endothelial cell proliferation.

**Fig. 5:**
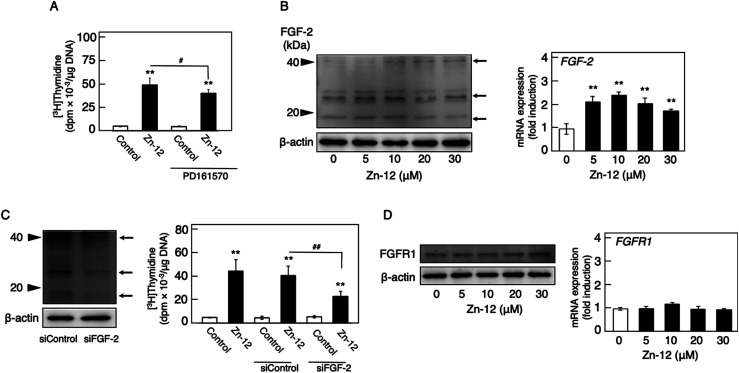
Involvement of the FGF-2 system in the stimulatory effect of Zn-12 on vascular endothelial cell proliferation. (A) The incorporation of [^3^H]thymidine into the acid-insoluble fraction of vascular endothelial cells. Growing cultures of bovine aortic endothelial cells were pretreated with or without PD161570 (1 μM) for 4 h and treated with Zn-12 (30 μM) for 24 h. The cells were labeled with [^3^H]thymidine during the last 4 h of the treatment. Values are means ± SE of four samples. *p < 0.05 *vs.* control; ^#^*p* < 0.05 *vs.* samples without PD161570. (B) The expression of FGF-2 protein (left panel) and FGF-2 mRNA (right panel) in vascular endothelial cells. Growing cultures of bovine aortic endothelial cells were treated with Zn-12 (5, 10, 20, or 30 μM) for 24 h (left panel) or 8 h (right panel). Values in the right panel are means ± SE of three samples. *p < 0.01 *vs.* control. Arrows in the left panel indicate the positions of FGF-2. (C) siRNA-mediated knockdown of endogenous FGF-2 (left panel) and its effect on the stimulation of the [^3^H]thymidine incorporation in vascular endothelial cells by Zn-12 (right panel). Growing cultures of bovine aortic endothelial cells were pretreated with siRNA against FGF-2 and then treated with or without Zn-12 (30 μM) for 24 h. The cells were labeled with [^3^H]thymidine during the last 4 h of the treatment. Values in the right panel are means ± SE of four samples. *p < 0.05 *vs.* control; ^#^*p* < 0.05 *vs.* samples without Zn-12. Arrows in the left panel indicate the positions of FGF-2. (D) The expression of FGFR1 protein (left panel) and FGFR1 mRNA (right panel) in vascular endothelial cells. Growing cultures of bovine aortic endothelial cells were treated with Zn-12 (5, 10, 20, or 30 μM) for 24 h (left panel) or 8 h (right panel). Values in the right panel are means ± SE of three samples.

Since endogenous FGF-2 is involved in the stimulation of vascular endothelial cells by inorganic zinc,^10^ the effect of the FGFR inhibitor PD161570 on the Zn-12-induced stimulation of [^3^H]thymidine incorporation in the cells was investigated (Fig. 5A). It was shown that the inhibitor partly but significantly reduced the stimulatory effect of Zn-12. The expression of the FGF-2 protein was not changed by Zn-12 treatment, although the FGF-2 mRNA level was significantly elevated by the zinc complex (Fig. 5B). siRNA-mediated knockdown of endogenous FGF-2 also partly but significantly reduced the stimulation of [^3^H]thymidine incorporation by Zn-12 (Fig. 5C). The expression of FGFR1 was not affected by Zn-12 at both the protein and mRNA levels (Fig. 5D). Taken together, these results suggest that the stimulation of vascular endothelial cells by Zn-12 depends on the endogenous FGF-2 signal pathway but does not require elevated expression levels of FGF-2 and FGFR1.

**Fig. 6:**
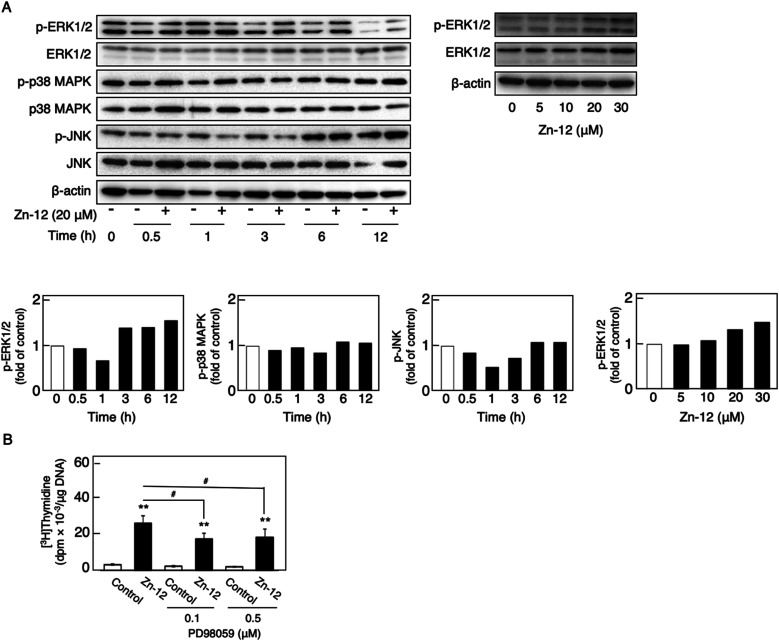
Involvement of the phosphorylation of MAPKs (ERK1/2, p38 MAPK, and JNK) in the stimulatory effect of Zn-12 on vascular endothelial cell proliferation. (A) Phosphorylation of MAPKs in vascular endothelial cells. Growing cultures of bovine aortic endothelial cells were treated with Zn-12 at 20 μM for 0.5, 1, 3, 6, and 12 h (upper left panel) or with Zn-12 at 5, 10, 20, and 30 μM for 6 h (upper right panel). The bands were assessed by densitometry; the bar graph shows the expression ratio of the phosphorylated MAPKs in the Zn-12-treated group compared with that in the control group at each time point or the phosphorylated ERK1/2 in the Zn-12-treated sample compared with that in the control sample (lower panels). (B) The incorporation of [^3^H]thymidine into the acid-insoluble fraction of vascular endothelial cells. Growing cultures of bovine aortic endothelial cells were pretreated with or without PD98059 (0.1 and 0.5 μM) for 4 h and treated with Zn-12 (20 μM) for 24 h. The cells were labeled with [^3^H]thymidine during the last 4 h of the treatment. Values are means ± SE of four samples. **p < 0.01 *vs.* control; ^#^*p* < 0.05 *vs.* samples without PD98059.

The phosphorylation of different MAPKs (ERK1/2, p38 MAPK, and JNK), downstream serine/threonine kinases that mediate FGF-2 signaling, in growing cultures of vascular endothelial cells was investigated after treatment with Zn-12. Zn-12 increased the phosphorylation of ERK1/2 but not that of p38 MAPK and JNK after 3 h and later (Fig. 6A, upper and lower left three panels). The effect of Zn-12 on ERK1/2 phosphorylation was concentration-dependent (Fig. 6A, upper and lower right panels). PD98059, a MEK1 inhibitor, partly but significantly reduced the stimulation of [^3^H]thymidine incorporation by Zn-12 (Fig. 6B), suggesting that the activation of ERK1/2 is partly involved in the stimulation of vascular endothelial cell proliferation by Zn-12.

## Discussion

**Fig. 7:**
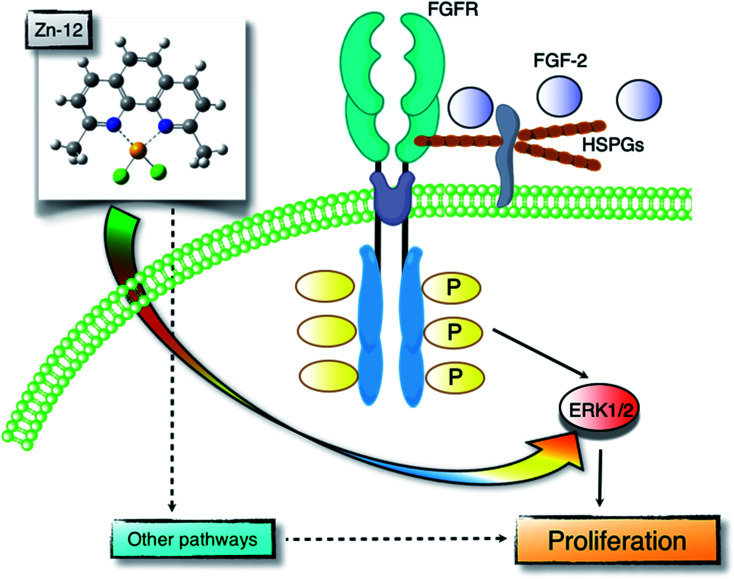
Zn-12 stimulates vascular endothelial cell proliferation. FGF-2 is one of the growth factors that promote vascular endothelial cell proliferation. FGF-2 forms a ternary complex with FGFR and heparan sulfate proteoglycans (HSPGs) and activates ERK1/2 to stimulate the proliferation. Stimulation of vascular endothelial cell proliferation by Zn-12 can be mediated by the ERK1/2 activation independently of the FGF-2–FGFR pathway. Additionally, there may be other pathways involved in the Zn-12 stimulation.

Proliferation of vascular endothelial cells is important for the repair process of damaged endothelial cell layers, a barrier between blood and subendothelial tissues. We have previously reported that the toxic heavy metal lead inhibits the proliferation of vascular endothelial cells and thus the repair process of damaged cell layers.^24,25^ The inhibitory effect was due to a lower response of the cells to endogenous FGF-2 as the expression of perlecan, a large heparan sulfate proteoglycan that promotes the binding of FGF-2 to FGFR, was decreased.^26,27^ In contrast, the essential trace element zinc stimulates vascular endothelial cell proliferation and promotes the repair during wound healing.^10,11^ This stimulation depends on endogenous FGF-2; however, zinc does not affect the expression of perlecan and the structure of heparan sulfate chains as well as the expression of FGF-2 and FGFR.^28^ It appears challenging to analyze the mechanisms underlying vascular endothelial cell proliferation using inorganic zinc as a molecular probe. Recently, from the viewpoint of toxicology, we have proposed bio-organometallics to analyze biological systems with organic–inorganic hybrid molecules as molecular probes.^12^ In the present study, we identified the Zn complex Zn-12 as a strong stimulator of vascular endothelial cell proliferation *in vitro*. The mechanisms underlying the Zn-12-mediated stimulation implicate the elevation of ERK1/2 phosphorylation, which mediates FGF-2 signaling to promote proliferation. This mechanism is only a part of the overall mechanism behind the Zn-12-induced stimulation; however, we postulate that the zinc complex is a promising molecular probe for analyzing biological systems for vascular endothelial cell proliferation (Fig. 7).

The structural characteristics of Zn-12 were investigated. It was demonstrated that the stimulatory activity of Zn-12 on vascular endothelial cell proliferation requires each constituent of the zinc complex and is independent of the intracellular content of zinc and iron. In other words, the combination of DMP with zinc atoms has a strong stimulatory effect on vascular endothelial cell proliferation, although the ligand alone lacks such activity and inorganic zinc exhibits only a weak effect. After comparing the structure of Zn-12 with those of Zn-11, Zn-13, Zn-14, Zn-15, Zn-19, and Zn-21, all of which have a phenanthroline structure, we postulate that methyl groups at the C2 and C9 positions play an important role in the stimulation of vascular endothelial cell proliferation. It is likely that these methyl groups contribute to the stability of Zn-12 as a coordinate compound by a steric effect, supporting the hypothesis that the whole structure of Zn-12 is required for the stimulation. The importance of the coordinate number was not observed in this study. On the other hand, we have previously reported that the cytotoxicity of organic–inorganic hybrid molecules is independent of the cytotoxicity of either the ligand alone or the intramolecular metal in its inorganic form.^18,22,23,29–32^ Activation of the NF-E2-related factor-2 by organic–inorganic hybrid molecules depends on the type of the coordinated metal ion.^33^ Thus, it seems that the biological activities of organic–inorganic hybrid molecules depend on the interaction between the ligand structure and the intramolecular metal. In fact, the three-dimensional molecular structure of organic–inorganic hybrid molecules can be dramatically changed by the intramolecular metal.^34^ This may be a reason why Zn-12 has a strong stimulatory effect on vascular endothelial cell proliferation. Although the chemical forms of Zn-12 accumulated within the cells are unknown, the intracellular accumulation of the zinc complex was almost the same as that of inorganic zinc, suggesting that the mechanisms by which Zn-12 stimulates vascular endothelial cell proliferation contain processes that differ from those by which inorganic zinc stimulates proliferation.

In the present study, we found that Zn-12 is a strong stimulator of vascular endothelial proliferation and revealed a part of the mechanisms underlying this stimulation. Specifically, the stimulation partly depends on the FGF-2 signal pathway, which is mediated by the phosphorylation of ERK1/2, without changing the expression levels of FGF-2 and FGFR1. Since both FGF-2 and Zn-12 increased the phosphorylation of ERK1/2 but not that of p38 MAPK and JNK, Zn-12 may mimic the function of FGF-2 to induce ERK1/2 phosphorylation. However, it was shown that this FGF-2 signal pathway-dependent mechanism is only a part of the overall mechanism underlying the stimulation of vascular endothelial cell proliferation by Zn-12. In other words, mechanisms other than FGF-2 signaling are mainly responsible for the stimulation. We have not yet identified these major mechanisms. However, we suggest that the target molecules of Zn-12, which are involved in the endogenous FGF-2-independent activation of ERK1/2, will be identified by experiments using Zn-12 as a molecular probe; meanwhile, other DMP metal complexes such as Mn-DMP, Ni-DMP, and Co-DMP may be used as inactive control probes for Zn-12. The present study supports the hypothesis that organic–inorganic hybrid molecules are promising molecular probes for analyzing biological systems.

## Conclusions

Bio-organometallics is a new strategy to analyze biological systems using organic–inorganic hybrid molecules as a molecular probe. In the present study, we identified a zinc complex, Zn(ii)2,9-dimethyl-1,10-phenanthroline (Zn-12), as a molecular probe to analyze the mechanisms underlying vascular endothelial cell proliferation. It was revealed that this zinc complex stimulates the proliferation by activating ERK1/2, downstream serine/threonine kinases that mediate FGF-2 signaling, without increasing the expression of FGF-2 and FGFR1. Our previous bio-organometallics studies and the present study contribute to the development of bio-organometallics.

## Conflicts of interest

There are no conflicts of interest to declare.

## Supplementary Material

RA-010-D0RA06731H-s001
