# Supplementary material for: Zn(ii)2,9-dimethyl-1,10-phenanthroline stimulates cultured bovine aortic endothelial cell proliferation
Source: RSC Adv. 2020 Nov 20;10(69):42327–37. doi: 10.1039/d0ra06731h (PMC9057965; doi:10.1039/d0ra06731h)
Supplement: RA-010-D0RA06731H-s001 [file RA-010-D0RA06731H-s001.pdf]

Electronic Supplementary Material (ESI) for RSC Advances.
This journal is © The Royal Society of Chemistry 2020

Table S1. Elemental analysis of metal complexes used in this study

Complex	Calcd C : H : N	Found C : H : N	Mach (%)	Complex	Calcd C : H : N	Found C : H : N	Mach (%)
Zn-1	15.74 : 4.84 : 6.12	15.51 : 4.84 : 6.03	98.19	Zn-21	41.07 : 2.01 : 7.98	40.83 : 2.30 : 7.84	95.02
Zn-2	35.18 : 4.92 : 20.51	34.90 : 4.68 : 20.43	97.97	Zn-22	44.56 : 4.22 : 11.99	44.64 : 4.13 : 12.03	99.12
Zn-3	29.39 : 3.21 : 6.86	29.18 : 3.07 : 6.73	97.67	Zn-23	39.07 : 2.93 : 19.53	38.76 : 3.08 : 19.81	97.64
Zn-4	22.50 : 3.78 : 13.12	21.90 : 3.72 : 12.86	97.74	Zn-24	40.78 : 3.41 : 9.51	40.56 : 3.61 : 9.34	97.38
Zn-5	44.40 : 3.42 : 0	44.30 : 3.66 : 0	96.61	Zn-25	64.28 : 3.32 : 5.77	64.23 : 3.36 : 5.73	99.35
Zn-6	43.08 : 3.46 : 4.19	43.00 : 3.69 : 4.12	97.30	Zn-26	Commercial product (>98%, TCI)		
Zn-7	37.75 : 3.52 : 9.78	37.50 : 3.48 : 9.68	99.06	Zn-27	45.56 : 5.35 : 0	45.39 : 4.98 : 0	96.36
Zn-8	42.95 : 3.90 : 12.52	43.05 : 3.73 : 12.54	98.42	Zn-28	Commercial product (>95%, TCI)		
Zn-9	41.06 : 2.76 : 9.58	41.12 : 2.96 : 9.55	97.59	Zn-29	25.85 : 5.21 : 12.06	25.82 : 4.75 : 11.71	96.05
Zn-10	48.75 : 3.00 : 11.37	48.47 : 3.10 : 11.14	98.06	Zn-30	21.54 : 7.83 : 25.12	21.56 : 7.41 : 24.97	97.98
Zn-11	45.54 : 2.55 : 8.85	45.56 : 2.83 : 8.85	96.69	Zn-31	Commercial product (>98%, TCI)		
Zn-12	48.80 : 3.51 : 8.13	48.72 : 3.72 : 8.27	97.50	Zn-32	Commercial product (>93%, TCI)		
Zn-13	47.24 : 3.05 : 8.47	47.43 : 3.00 : 8.44	99.20	Mn-DMP	50.33 : 3.62 : 8.38	50.07 : 3.80 : 7.90	96.34
Zn-14	51.57 : 4.33 : 7.52	51.49 : 4.23 : 7.47	98.96	Fe-DMP	48.88 : 3..81 : 8.14	49.13 : 4.21 : 7.68	94.79
Zn-15	41.07: 2.01 : 7.89	40.95 : 2.12 : 7.98	97.80	Co-DMP	49.73 : 3.58 : 8.29	49.47 : 3.65 : 7.91	97.66
Zn-16	60.06 : 4.12 : 6.37	59.82 : 4.03 : 6.25	98.51	Ni-DMP	48.48 : 3.78 : 8.08	48.07 : 3.69 : 8.06	98.86
Zn-17	62.15 : 3.91 : 6.04	61.97 : 3.90 : 6.08	99.60	Cu-DMP	49.06 : 3.53 : 8.17	49.03 : 3.57 : 8.07	99.20
Zn-18	71.57 : 7.67 : 4.64	71.40 : 7.49 : 4.49	98.06	Cd-DMP	42.94 : 3.09 : 7.15	42.82 : 3.14 : 6.98	98.58
Zn-19	48.80 : 3.51 : 8.13	48.63 : 3.42 : 8.04	98.66	Hg-DMP	35.05 : 2.52 : 5.84	34.92 : 2.55 : 5.76	99.03
Zn-20	59.58 : 4.58 : 5.79	59.25 : 4.30 : 5.76	97.61	Pb-DMP	31.17 : 2.24 : 10.39	31.37 : 2.32 : 10.09	97.67

Table S1
